# Editorial: Microbiota in skin inflammatory diseases

**DOI:** 10.3389/fimmu.2023.1235314

**Published:** 2023-06-15

**Authors:** Eun Jeong Park, Hariom Yadav, Tej Pratap Singh

**Affiliations:** ^1^ Department of Molecular Pathobiology and Cell Adhesion Biology, Mie University Graduate School of Medicine, Tsu, Japan; ^2^ Center for Microbiome Research, Microbiomes Institute, Morsani College of Medicine, University of South Florida, Tampa, FL, United States; ^3^ Department of Pathobiology, University of Pennsylvania, Philadelphia, PA, United States

**Keywords:** skin, microbiota, inflammation, metagenomics, psoriasis, atopic dermatitis

Mammalian skin furnishes a niche habitat to diverse microorganisms. The skin contains hair follicles or appendages that provide the home presumably favorable for microbial accessibility (1). Various microbes, including fungi, bacteria, and viruses, inhabit the interface between the skin and external area to constitute the microbiota (2). This microbiota of skin is quite analogous to that of the gut in terms of developing homeostasis and the immune system (3). Microbiota interactions with epithelial or immune cells play critical roles in wound healing, barrier restoration, or pathogenic protection (4). The quantitative and qualitative changes in skin microbiota, such as dysbiosis, may closely associate with the dysfunction of the host immune system, possibly leading to the induction of inflammatory disorders. Although the skin microbiota might play crucial roles in remodeling host immune and inflammatory responses, the exact mechanisms by which the alterations in skin microbiota induce inflammatory disorders need to be defined. Thus, it is imperative to systemically explore the etiologic factors in charge of compositional changes in skin microbiota. For instance, as for the approach to uncover the unknown mechanisms responsible for these changes, it is essential to validate cellular and molecular interactions between skin microbiota and host cells, which are helpful to better understand the cause and effect of the pathogenesis of skin inflammations such as psoriasis and atopic dermatitis (AD). Such approaches may provide insight into further developing diagnostics and therapeutics for skin inflammations. The current Research Topic encompasses some recent findings related to the roles of cutaneous microbiota in affecting the pathophysiology of inflammatory diseases. In a comprehensive review, Chen et al. overviewed methodological advances in analyzing the skin microbiome, significant challenges in systemically substantiating the skin microbial communities, and current knowledge of skin microbiome in health and disease. Moreover, the authors comprehensively discussed the reports examining the microbiome characteristic of diverse skin diseases.

A recent study highlighted the roles played by the skin-resident immune cells in modifying the progression of microbiota-involved infectious diseases. Park et al. have shown that the gut microbiota is critical in the induction of CD169^+^ skin macrophages associated with the signaling of type I interferon. This cutaneous macrophage subset, relying on the gut microbiota, can protect mice from *Staphylococcus aureus* skin infection. This protective function required the recruitment and activation of dermal γδ T cells *via* interleukin (IL)-23. Park et al.'s study has provided a molecular basis for a better understanding of the potential role of gut microbiota in the induction of remote CD169^+^ skin macrophages to affect bacterial skin infection. Pemphigus vulgaris (PV) is an autoimmune disease characterized by blisters and erosions on the skin and mucous membranes (5, 6). A recent study reported the alterations in gut microbiota composition and plasma cytokine levels in PV (7), which may require subsequent studies for validation. Wang et al. have examined the changes in gut microbiota and metabolites on the onset and treatment of PV using metagenomics and metabolomic tools. The increase in the pathogenic bacteria and decrease in the probiotics producing short chain fatty acids (SCFA) were shown to correlate with PV onset.

From a possible genetic contribution to AD onset, examining the associations between gut microbiota compositions and maternal influences on developing AD in children is essential. Fan et al. found that pediatric AD development correlated with the enrichment or reduction of particular microbes in mothers’ gut during pregnancy. Thus, Fan et al.'s research offers a possible way to manipulate gut microbiota in pregnant mothers possibly to prevent AD in their offspring. Environmental factors are the driver in modifying the skin microbial composition, possibly through eliciting dysbiosis of particular bacteria. Some pollutants can restrict ceramide production mediated by skin *Roseomonas mucosa* and thus drive its dysbiosis during atopic dermatitis (8). Gough et al. investigated the effects of *R. mucosa* in psoriasis-like skin inflammation. Treatment of this microbe affected tumor necrosis factor signaling in cultured keratinocytes and alleviated symptoms in a mouse model of psoriasis. The authors further observed a dependable correlation between psoriatic severity and the influence of environmental factors such as carbon monoxide. Gough et al.'s works show a potential benefit of microbial manipulation and ecological mitigation for reducing the inflammatory responses, presumably in AD and psoriasis. Head and neck dermatitis (HND), a phenotypic variant of AD, often accompanied with facial erythema, remains therapeutically challenging (9). Due to more sensitivity to *Malassezia furfur* than other AD patients, HND patients showed improved symptoms upon antifungal treatment (10). Chu et al. examined the underlying mechanisms by which the pathogenesis of HND is associated with *M. furfur*. The levels of ceramide were reduced, while the proinflammatory mediators and T-helper 2 type cytokines were increased with *M. furfur*, revealing the effects of this fungal microbe on contributing to HND pathogenesis.

Tissue-specific microbiota contributes to intrinsic tissue homeostasis. Schwarz et al. have demonstrated the potential roles of the skin microbiota in controlling inflammation in a mouse model of psoriasis. The effect of topical treatment with SCFA on ameliorating inflammatory responses in psoriasis can be derived from the signaling of SCFA across hydroxycarboxylic acid receptor 2 (HCA2). Schwarz et al. sought to elucidate the role of HCA2 in inducing regulatory T (Treg) cells to downregulate skin inflammation. The HCA2-knockout (KO) mice showed an exaggerated inflammation due to a decline of Treg cells from a remarkable change in skin microbiota. The HCA2-KO mice recovered functional activity of the Treg cells to suppress inflammatory responses in psoriasis, upon being co-housed with WT mice, due to the restoration of the skin microbiota in the KO mice. This study gave us insight into the potential impacts of modifying the skin microbial composition on alleviating cutaneous inflammations. Luo et al. shed light on the novel roles of butyrate produced by *Staphylococcus epidermidis* in alleviating inflammatory symptoms aggravated by infection with *S. aureus* in an established AD-like model. Specifically, butyrate can downregulate dermal IL-33 expression and leukocyte infiltration and attenuate skin inflammation by inhibiting histone deacetylase 3. Thus, this study provides scientific evidence on butyrate as a potential agent to treat skin inflammations in AD patients. Joshi et al. discussed potential AD therapeutics of microbe-derived antimicrobial peptides (AMP). Because the reduced AMP levels in AD patients derived from the increased colonization of *S. aureus*, the authors pointed out the significance of the selective killing of *S. aureus* without impairment of the commensal microbiome as a therapeutic future avenue. *S. epidermidis* is an abundant bacterium that inhabits healthy human skin, but *S. epidermidis* can also be harmful to the skin, for example inducing AD, depending on the strains (11). Landemaine et al. characterized 12 strains of *S. epidermidis* isolated from healthy and AD skin. The strains from AD skin displayed three functional features: i) structural modification of the epidermis in a model of a 3D reconstructed skin; ii) no induction of aryl hydrocarbon receptor/ovo-like1 pathway to limit production of indole metabolites in co-cultures with normal human epidermal keratinocytes; and consequently, iii) alteration in skin differentiation markers such as desmoglein-1 and filaggrin. This study offers insight into how *S. epidermidis* communicates with the skin in health and disease.

Narrowband ultraviolet-B (nbUVB) phototherapy has been broadly utilized as a dermatological therapy effective in psoriasis, AD, and other inflammatory dermatitis (12–14). Hooper et al. performed a longitudinal analysis of skin microbiota in cutaneous T-cell lymphoma (CTCL) patients treated with nbUVB. The authors probed changes in skin microbiota composition *via* 16S rRNA sequencing analysis. The nbUVB treatment increased the microbial complexity and decreased two pathogens *S. aureus* and *S. lugdunensis*, in the skin of CTCL patients. This study provides a global implication to gauge a biological assessment of nbUVB-induced changes in the skin microbiome. On the other hand, radiation-induced skin injury (RISI) can occur after radiotherapy. Huang et al. investigated the composition and function of skin microbiota changed by RISI. Although further validation requires identifying its interactive functions with skin, the phylum Firmicutes, considered active for wound healing, was predominantly upregulated in RISI. This study proposes the microbiota and metabolites as potential targets for RISI therapeutics.

We have cited and summarized just a part of the papers chosen depending on the intent of the Research Topic. Thus, we earnestly apologize that a majority of original and review articles are not included in the references due to the restricted capacity of this Editorial.

## Author contributions

All authors listed conceived the idea, contributed to preparing the draft, edited the manuscript, and approved the work for publication.
